# Supplementary material for: Metabolic and transcriptomic analyses of nectaries reveal differences in the mechanism of nectar production between monocots (Ananas comosus) and dicots (Nicotiana tabacum)
Source: BMC Plant Biol. 2024 Oct 9;24:940. doi: 10.1186/s12870-024-05630-3 (PMC11462711; doi:10.1186/s12870-024-05630-3)
Supplement: Supplementary file 1 — Supplementary Material 1 [file 12870_2024_5630_MOESM1_ESM.pdf]

Supplementary Material

Metabolic and transcriptomic analyses of nectaries reveal differences in the mechanism of nectar production between monocots (*Ananas comosus*) and dicots (*Nicotiana tabacum*)

Author: Thomas Göttlinger*, Marcello Pirritano, Martin Simon, Janina Fuß, Gertrud Lohaus

*Correspondence: Thomas Göttlinger (goettlinger@uni-wuppertal.de)

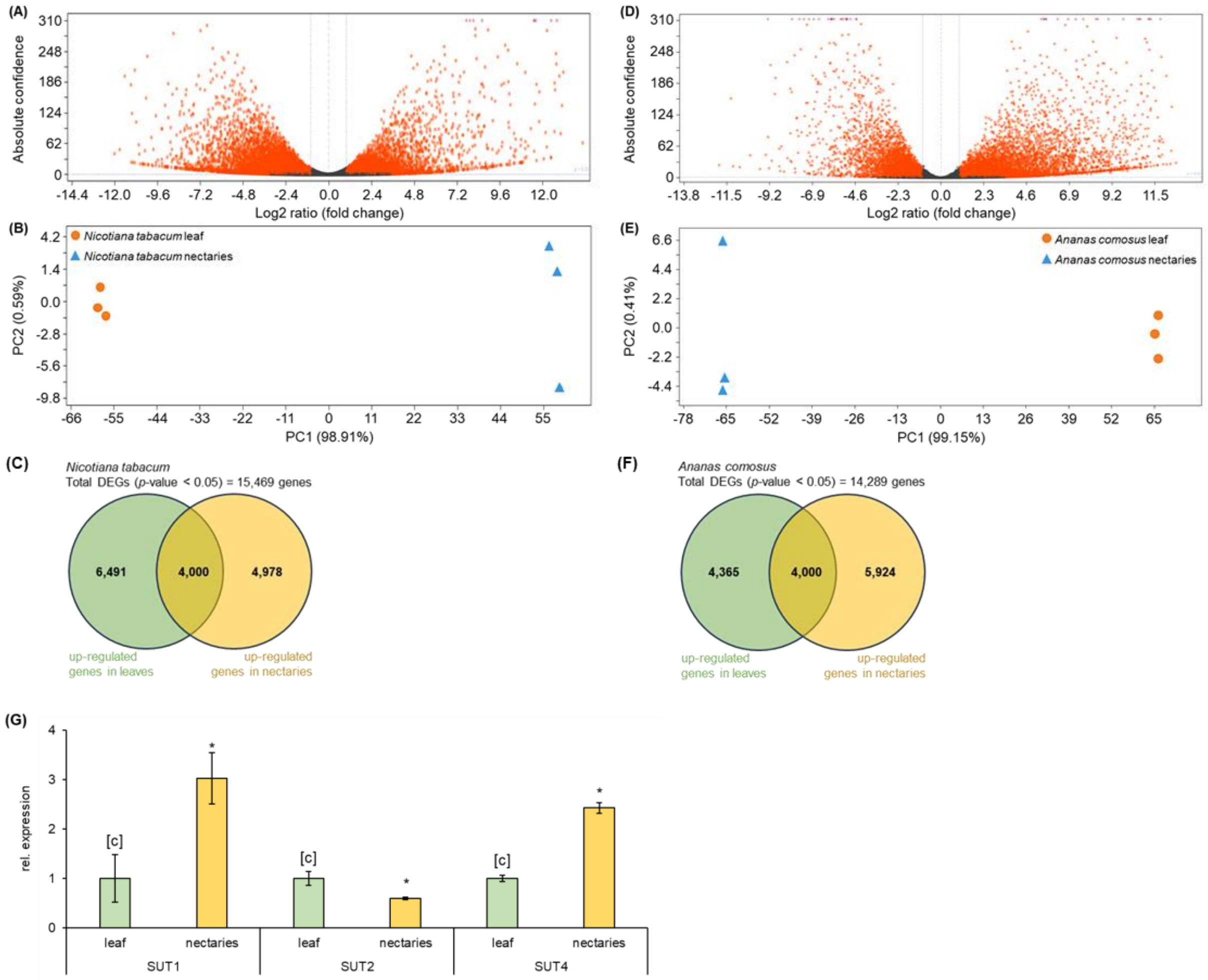

Supplementary Figure S1: **Overall gene expression profiles in the nectaries and leaves of *Nicotiana tabacum* and *Ananas comosus* and validation of transcriptomic data.**
The Volcano plot (A: *N. tabacum*, D: *A. comosus*) is generated by using expression levels using DESeq2 and shows the most highly differentially expressed loci. In this graphical presentation, fold change (log2 ratio) is plotted against absolute confidence (-log10 adjusted *p*-value). Each gene is represented by one dot and the orange dots have a *p*-value smaller than 0.05 in this plot and are the most differentially expressed genes. The red asterisks are genes with a *p*-value of zero. Principal component analysis (PCA) was used to visualize the variation between expression analysis samples (B: *N. tabacum*, E: *A. comosus*). The first principal component (PC 1) describes about 99 % and the second principal component (PC 2) describes less than 1 % of the dataset variation (B, E). In the Venn diagram, the DEGs (*p*-value < 0.05) are divided into up-regulated genes in leaves (green) and up-regulated genes in nectaries (yellow) for *Nicotiana tabacum* (C) and *Ananas comosus* (F). The number of genes in the overlapping of the two circles represents the number of genes that are neither up-regulated nor down-regulated and therefore cannot be clearly assigned to a tissue (C, F). Relative expressions of sucrose transporters SUT1, SUT2, and SUT4 in leaves and nectaries of *Ananas comosus* (G). Samples of leaves (n=3) were set as calibrator [c] for each sucrose transporter in comparison to nectaries (n=3). Student's t-test was performed to mark significant differences by asterisks (*p*-value < 0.05).

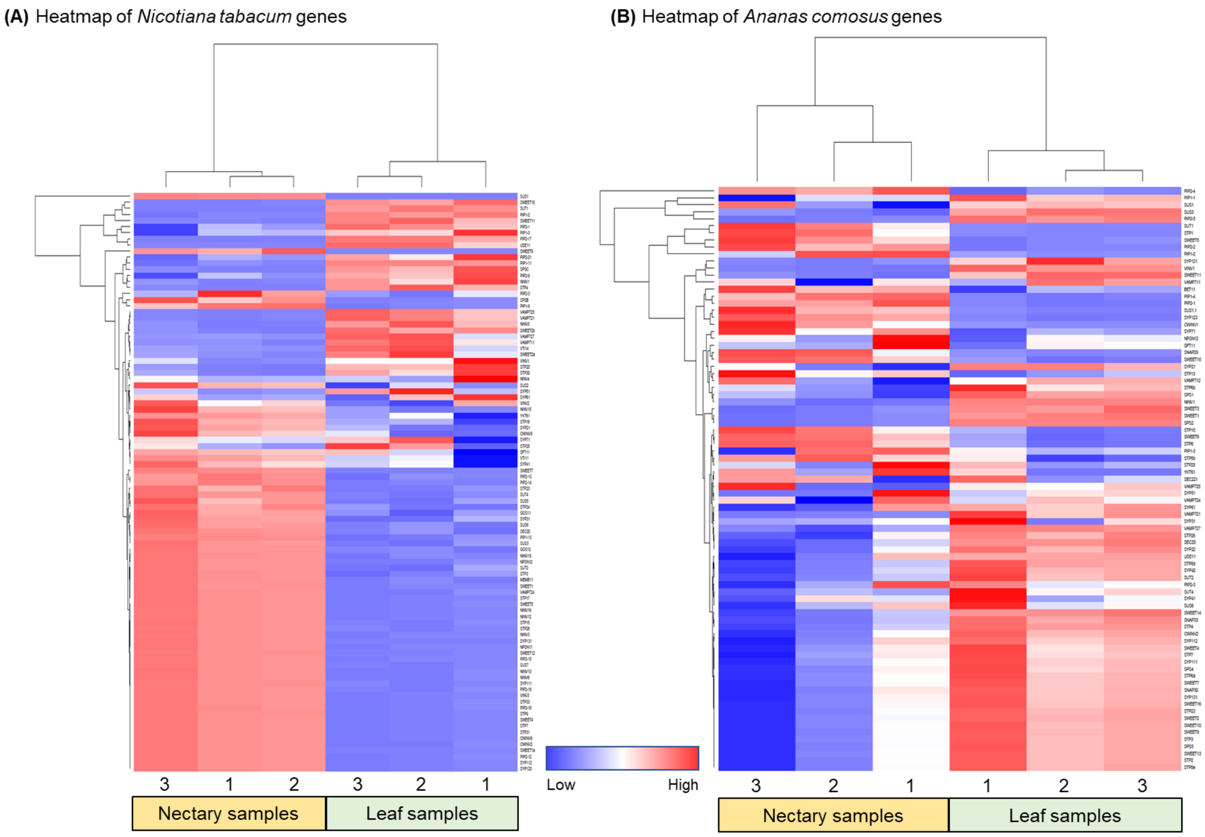

Supplementary Figure S2: Expression heatmaps of the *Nicotiana tabacum* (A) and *Ananas comosus* (B) genes.

The heatmap shows the scaled TPM values of different genes (SPS, INV, SUS, SUT, SWEET, STP, PIP, SNARE) of the individual samples of leaves and nectaries.

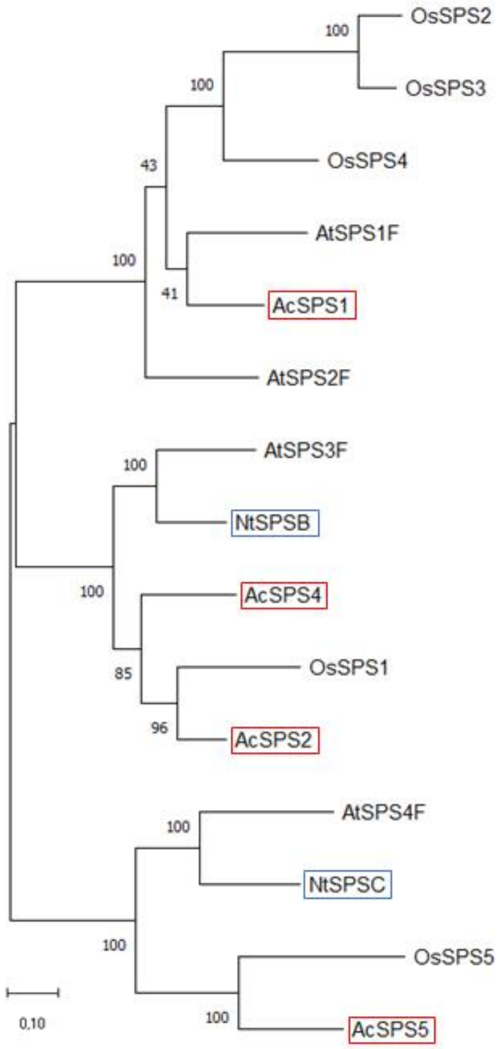

Supplementary Figure S3: **Phylogenetic analysis of selected sucrose phosphate synthases (SPS).** Phylogenetic analysis was carried out with the species *A. thaliana*, *N. tabacum*, *O. sativa*, and *A. comosus*. Protein alignment of the sucrose phosphate synthases was carried out by ClustalW. A maximum likelihood tree with 1,000 bootstrap iterations was calculated. Bar indicates evolutionary distance; numbers indicate percentage of bootstrap analysis. The red frame highlights the pineapple genes and the blue frame highlights the tobacco genes. Gene IDs are in Supplementary Table S9-S12.

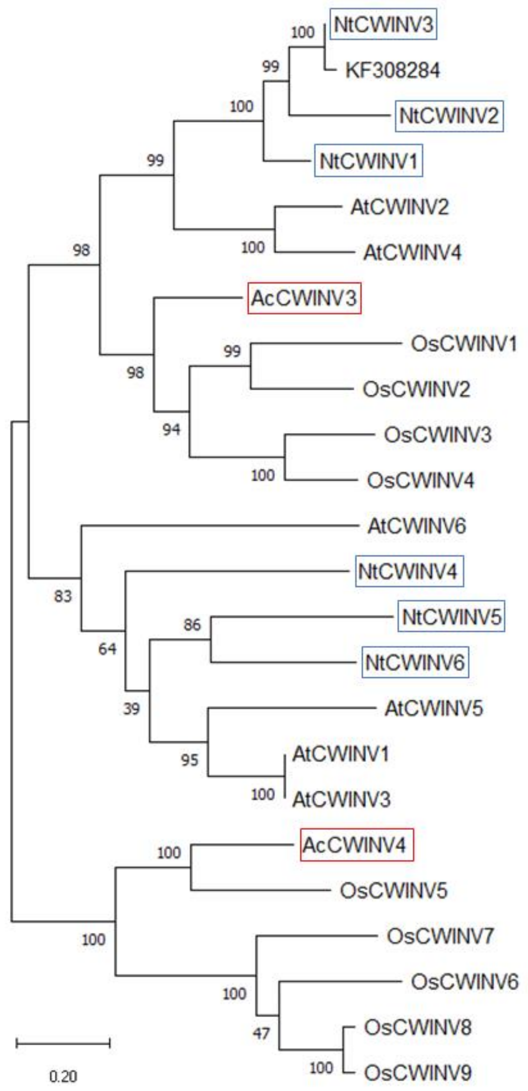

Supplementary Figure S4: **Phylogenetic analysis of selected cell wall invertases (CWINV).** Phylogenetic analysis was carried out with the species *A. thaliana*, *N. tabacum*, *O. sativa*, and *A. comosus*. Protein alignment of the invertases was carried out by ClustalW. A maximum likelihood tree with 1,000 bootstrap iterations was calculated. Bar indicates evolutionary distance; numbers indicate percentage of bootstrap analysis. The red frame highlights the pineapple genes and the blue frame highlights the tobacco genes. Gene IDs are in Supplementary Table S9-S12.

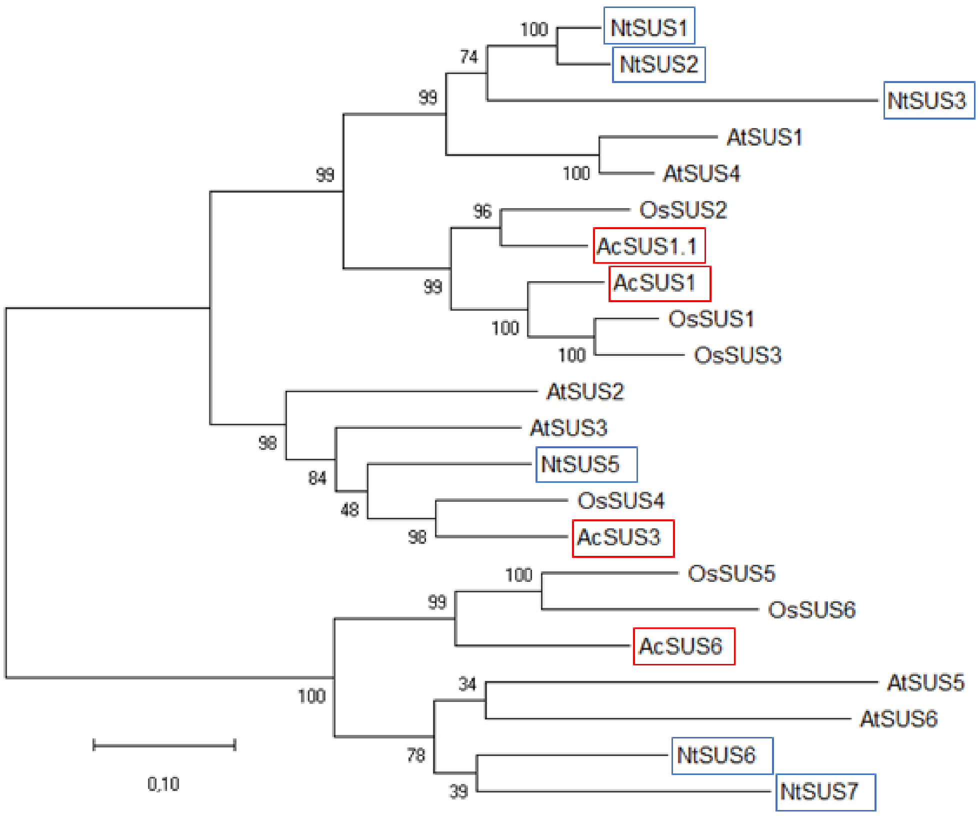

Supplementary Figure S5: **Phylogenetic analysis of selected sucrose synthases (SUS).** Phylogenetic analysis was carried out with the species *A. thaliana*, *N. tabacum*, *O. sativa*, and *A. comosus*. Protein alignment of the sucrose synthases was carried out by ClustalW. A maximum likelihood tree with 1,000 bootstrap iterations was calculated. Bar indicates evolutionary distance; numbers indicate percentage of bootstrap analysis. The red frame highlights the pineapple genes and the blue frame highlights the tobacco genes. Gene IDs are in Supplementary Table S9-S12.

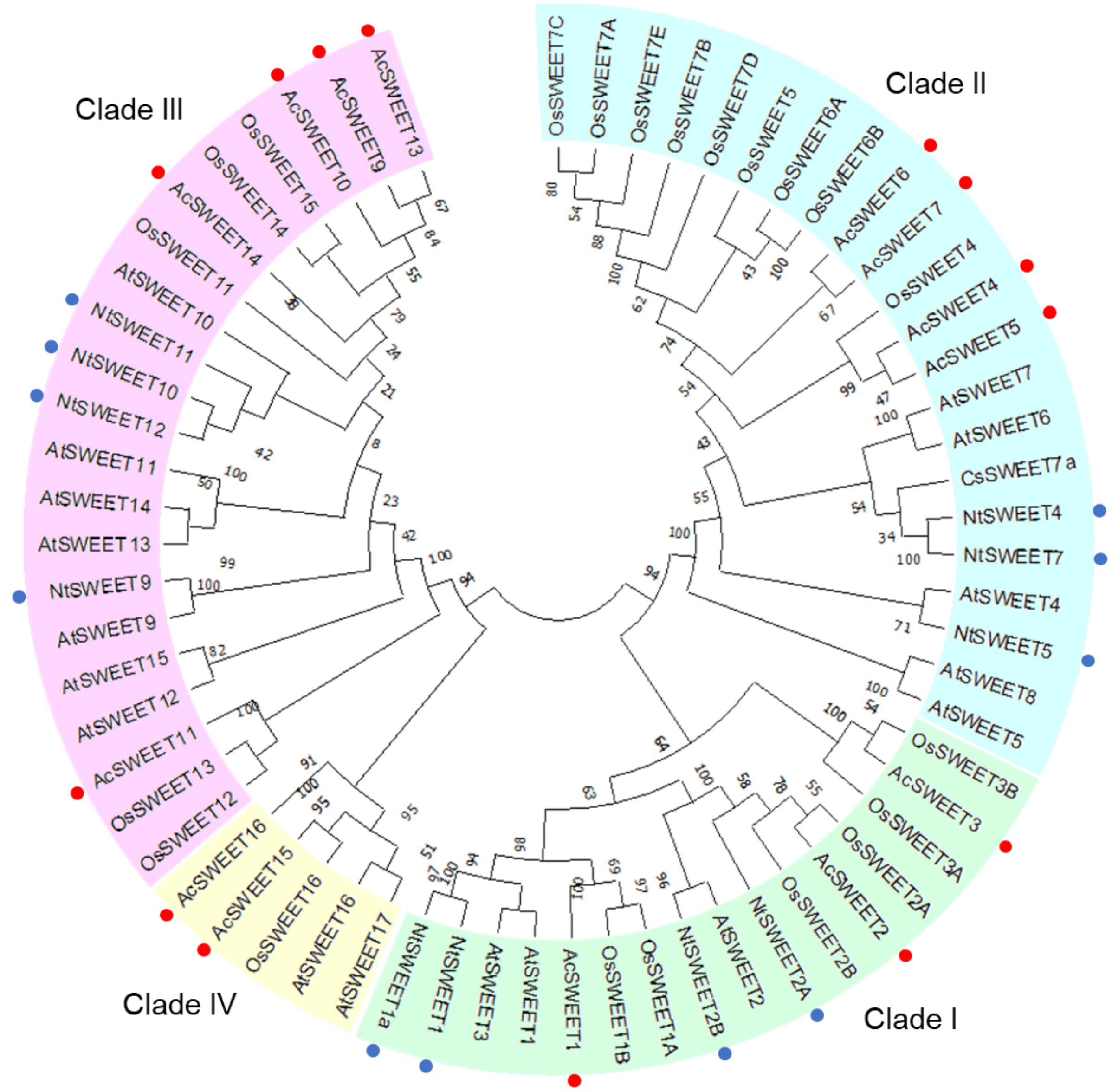

Supplementary Figure S6: **Phylogenetic analysis of selected sugars will eventually be exported transporters (SWEET).**

Phylogenetic analysis was carried out with the species *A. thaliana*, *N. tabacum*, *O. sativa*, and *A. comosus*. In addition, the sugar transporter CsSWEET7a was also used for this purpose, due to its role in the unloading of the apoplasmic phloem into nectar during cucumber anthesis (Li et al., 2021). Protein alignment of the transporters was carried out by ClustalW. A maximum likelihood tree with 1,000 bootstrap iterations was calculated. Bar indicates evolutionary distance; numbers indicate percentage of bootstrap analysis. SWEETs are divided into different clades depending on Lin *et al.*, 2022. The red point highlights the pineapple genes and the blue point highlights the tobacco genes. Gene IDs are in Supplementary Table S9-S12.

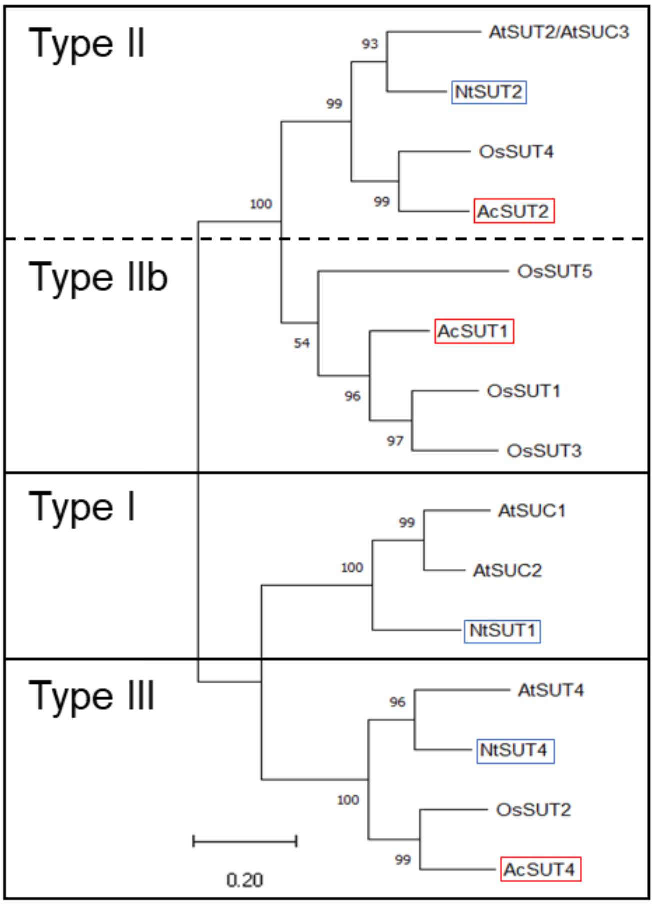

Supplementary Figure S7: **Phylogenetic analysis of selected sucrose uptake transporters (SUT).** Phylogenetic analysis was carried out with the species *A. thaliana*, *N. tabacum*, *O. sativa*, and *A. comosus*. Protein alignment of the sucrose transporter was carried out by ClustalW. A maximum likelihood tree with 1,000 bootstrap iterations was calculated. Bar indicates evolutionary distance; numbers indicate percentage of bootstrap analysis. SUTs are divided into different clades or types depending on Sauer 2007 and Peng et al. 2014. The red frame highlights the pineapple genes and the blue frame highlights the tobacco genes. Gene IDs are in Supplementary Table S9-S12.

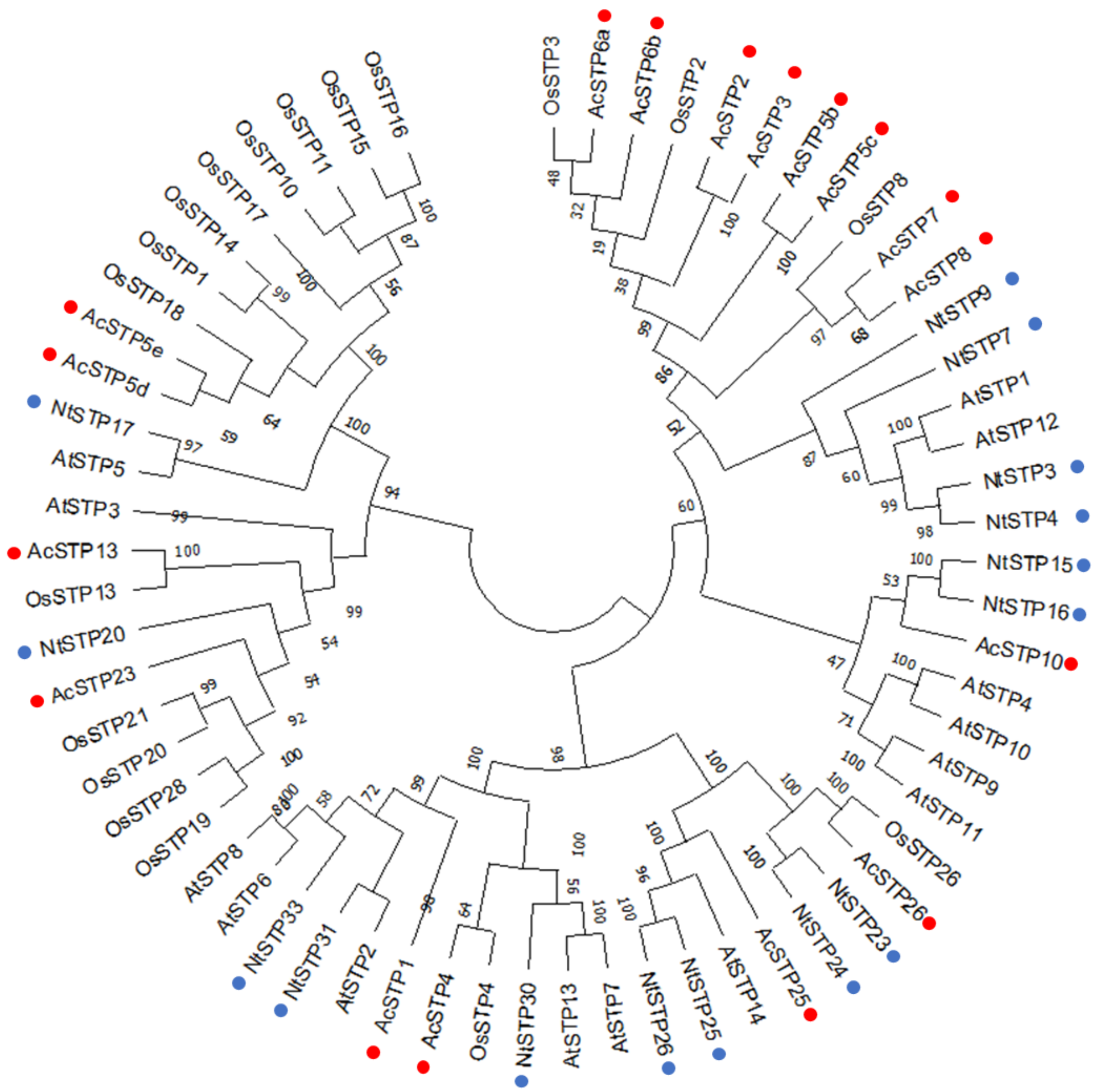

Supplementary Figure S8: **Phylogenetic analysis of selected sugar transport proteins (STP).** Phylogenetic analysis was carried out with the species *A. thaliana*, *N. tabacum*, *O. sativa*, and *A. comosus*. Protein alignment of the transport proteins was carried out by ClustalW. A maximum likelihood tree with 1,000 bootstrap iterations was calculated. Bar indicates evolutionary distance; numbers indicate percentage of bootstrap analysis. The red point highlights the pineapple genes and the blue point highlights the tobacco genes. Gene IDs are in Supplementary Table S9-S12.

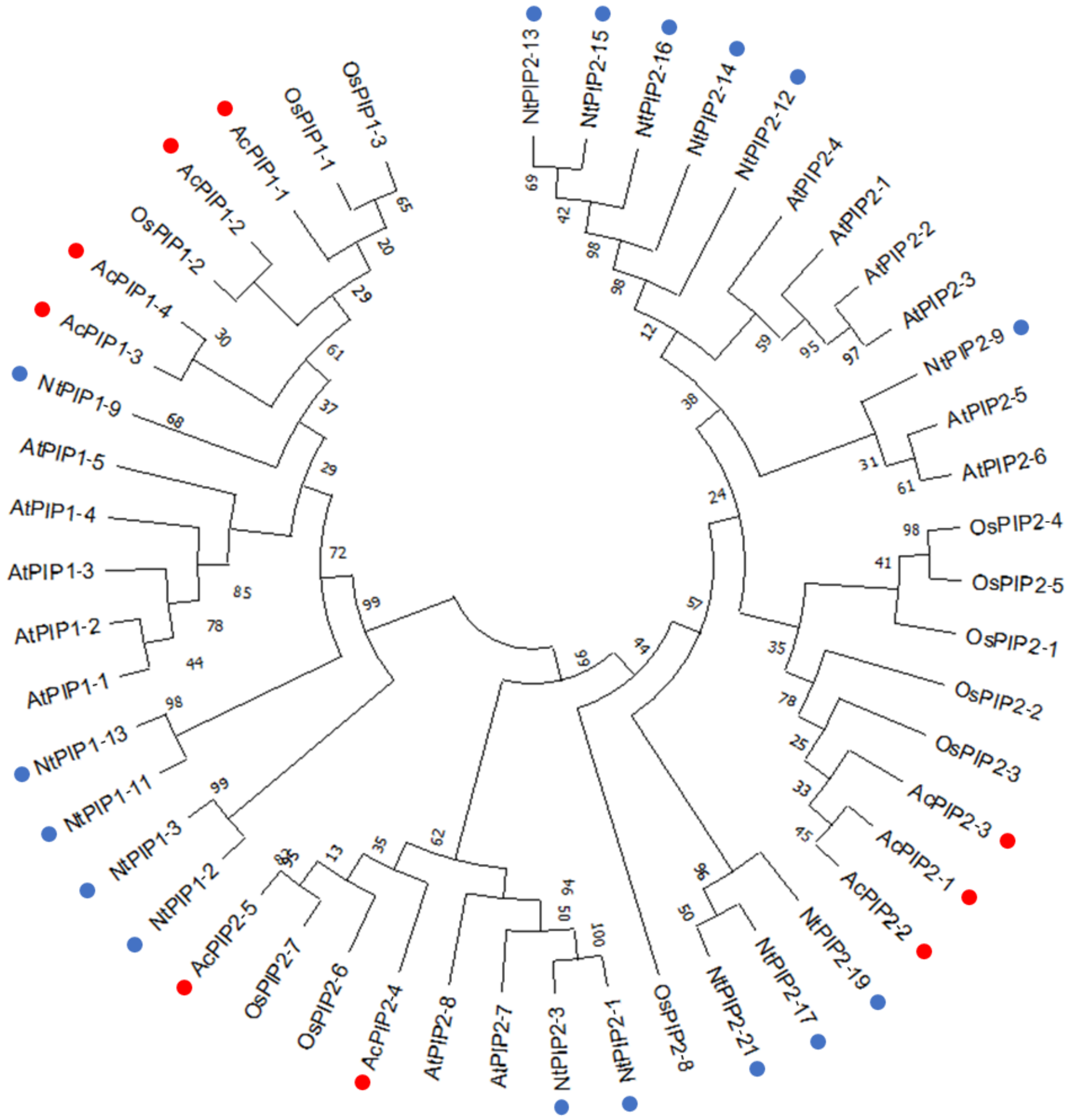

Supplementary Figure S9: **Phylogenetic analysis of selected plasma membrane intrinsic proteins (PIP).** Phylogenetic analysis was carried out with the species *A. thaliana*, *N. tabacum*, *O. sativa*, and *A. comosus*. Protein alignment of the intrinsic proteins was carried out by ClustalW. A maximum likelihood tree with 1,000 bootstrap iterations was calculated. Bar indicates evolutionary distance; numbers indicate percentage of bootstrap analysis. The red point highlights the pineapple genes and the blue point highlights the tobacco genes. Accession numbers are in Supplementary Table S9-S12.

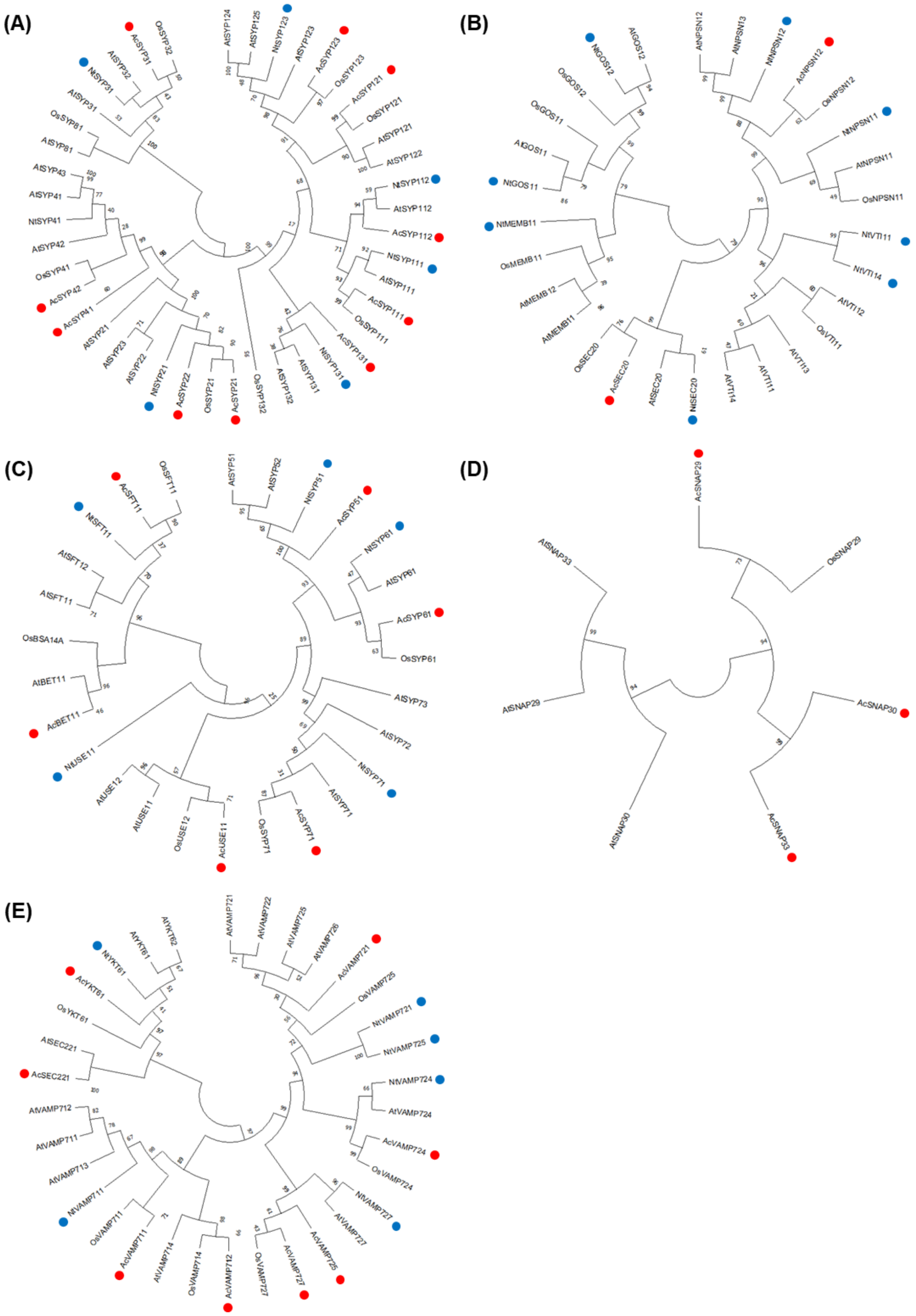

Supplementary Figure S10: **Phylogenetic analysis of selected soluble *N*-ethylmaleimide-sensitive-factor attachment receptor (SNARE)-domain-containing proteins.**
Phylogenetic analysis was carried out with the species *A. thaliana*, *N. tabacum*, *O. sativa*, and *A. comosus*. A separate phylogenetic tree was created for each clade (A: Clade Qa; B: Clade Qb; C: Clade Qc; D: Clade Qb + Qc; E: Clade R). Protein alignment of the proteins was carried out by ClustalW. A maximum likelihood tree with 1,000 bootstrap iterations was calculated. Bar indicates evolutionary distance; numbers indicate percentage of bootstrap analysis. The red point highlights the pineapple genes and the blue point highlights the tobacco genes. Gene IDs are in Supplementary Table S9-S12.

Supplementary Table S1: Water content in nectaries and leaves, expressed as a percentage of fresh weight.

Species	Nectaries [%]	Leaves [%]
Nicotiana tabacum	75.4 ± 1.7	85.6 ± 1.6
Ananas comosus	75.1 ±1.5	80.1 ± 1.7

Supplementary Table S2: Metabolic and enzymatic data of *Nicotiana tabacum*.

	sugar [mM]					invertase [U/g FW]			
**tissue**	**glucose**	**fructose**	**sucrose**	**sum amino acids [mM]**	**starch [mg/g FW]**	**cell wall**	**vacuolar**	**neutral**	**SUS [U/g FW]**
Leaf	5.1	2.6	21.5	9.2	6.5	-	-	-	-
Leaf	2.9	1.4	16.7	8.9	25.6	-	-	-	-
Leaf	3.0	1.1	20.2	11.0	48.5	-	-	-	-
Leaf	1.6	1.0	20.7	11.5	51.0	-	-	-	-
Nectaries	196.1	196.1	184.8	81.7	2.1	41.6	2.9	0.3	4.5
Nectaries	141.7	141.7	215.3	175.4	1.8	24.3	1.1	0.6	3.9
Nectaries	218.2	218.2	133.1	121.6	1.1	28.9	1.0	1.3	3.1
Nectaries	127.3	127.3	130.1	202.6	3.0	-	-	-	-
Nectar	621.3	642.7	289.6	1.4	0.0	-	-	-	-
Nectar	270.2	296.1	236.0	1.1	0.0	-	-	-	-
Nectar	456.7	363.7	184.9	0.7	0.0	-	-	-	-
Nectar	330.1	405.2	156.2	0.6	0.0	-	-	-	-

Supplementary Table S3: Metabolic and enzymatic data of *Ananas comosus*.

	sugar [mM]					invertase [U/g FW]			
tissue	glucose	fructose	sucrose	sum amino acids [mM]	starch [mg/g FW]	cell wall	vacuolar	neutral	SUS [U/g FW]
Leaf	8.6	10.9	40.9	5.8	5.3	-	-	-	-
Leaf	1.8	4.3	33.1	4.4	16.6	-	-	-	-
Leaf	14.4	12.9	41.2	2.8	10.0	-	-	-	-
Leaf	15.2	11.8	12.8	1.8	8.1	-	-	-	-
Nectaries	18.8	21.7	37.3	1.9	-	-	-	-	-
Nectaries	90.9	109.7	57.8	35.7	1.5	4.8	1.0	3.4	0.8
Nectaries	110.1	130.2	103.2	28.2	2.0	4.9	1.6	1.2	1.0
Nectaries	142.5	142.5	48.7	44.6	2.4	4.3	0.7	1.8	0.8
Nectar	109.8	109.8	31.8	30.8	2.2	-	-	-	-
Nectar	162.3	162.3	40.8	33.0	-	-	-	-	-
Nectar	367.9	258.8	204.2	2.6	0.0	-	-	-	-
Nectar	353.8	218.4	161.9	1.9	0.0	-	-	-	-

Supplementary Table S4: Total reads of different samples of *Ananas comosus* and *Nicotiana tabacum*

Species	Tissue	Total reads after trimming
Nicotiana tabacum	Leaf	107.1 million reads
	Leaf	57.1 million reads
	Leaf	71.2 million reads
	Nectary	66.8 million reads
	Nectary	66.3 million reads
	Nectary	55.9 million reads
Ananas comosus	Leaf	39,5 million reads
	Leaf	73.2 million reads
	Leaf	60.1 million reads
	Nectary	59.6 million reads
	Nectary	54.4 million reads
	Nectary	53.9 million reads

Supplementary Table S5: TPM values of different genes in *Nicotiana tabacum*.

	leaf			nectary		
**gene name**	**TPM1**	**TPM2**	**TPM3**	**TPM1**	**TPM2**	**TPM3**
SPSB	6.7	4.4	4.3	254.9	389.1	652.7
SPSC	79.7	47.9	55.7	7.3	11.1	17.7
NINV1	49.7	28.2	35.6	12.9	21.1	29.5
NINV3	2.0	1.9	1.7	4.8	5.3	6.3
NINV4	25.0	14.2	15.7	16.1	20.1	28.5
NINV5	34.8	35.0	32.2	43.1	52.0	58.8
NINV6	1.6	0.7	1.0	6.7	8.8	12.5
NINV12	1.5	1.2	1.3	0.1	0.1	0.1
NINV13	1.6	0.7	1.0	6.7	8.8	12.5
NINV15	16.7	14.0	15.1	31.1	34.1	40.8
NINV16	1.5	1.2	1.3	0.1	0.1	0.1
NINV19	4.4	4.2	3.8	7.0	7.7	9.0
CWINV2	0.3	0.0	0.2	0.0	0.2	0.1
CWINV5	12.8	10.9	12.7	0.0	0.2	0.3
CWINV6	0.0	0.0	0.0	0.0	0.1	0.1
VINV1	32.2	20.0	21.1	38.9	44.7	75.4
VINV2	20.1	13.6	14.8	30.8	40.0	53.9
VINV3	0.7	0.6	0.5	0.2	0.4	0.4
SUS1	11.9	13.1	10.8	4154.3	4239.7	5700.1
SUS2	17.2	16.5	13.4	64.0	65.6	103.2
SUS3	8.0	5.6	6.3	4.5	5.8	7.0
SUS5	12.3	8.7	9.6	33.1	53.1	79.8
SUS6	8.2	7.9	7.8	7.3	12.0	14.5
SUS7	1.1	0.7	1.1	3.9	5.7	7.1
SWEET1	2.9	2.8	2.5	3.8	9.9	9.4
SWEET1a	0.2	0.0	0.2	0.2	0.1	0.0
SWEET2a	22.3	28.0	26.2	21.7	20.6	24.1
SWEET2b	34.5	27.1	32.2	11.4	10.9	11.2
SWEET4	0.0	0.0	0.0	1.9	3.6	4.7
SWEET5	1.2	1.0	0.9	0.2	0.4	1.7
SWEET7	0.0	0.2	0.0	129.8	129.8	180.2
SWEET9	0.1	0.1	0.3	857.4	1308.9	1311.0
SWEET10	255.6	168.1	190.6	49.1	79.3	76.7
SWEET11	152.7	178.2	163.7	6.7	7.7	9.9
SWEET12	0.7	0.6	1.0	9.7	13.1	14.1
SUT1	215.3	196.6	179.0	90.6	90.1	119.0
SUT2	5.3	2.1	2.7	1.2	1.9	2.5
SUT4	8.5	6.4	7.4	41.1	46.7	62.2
STP3	4.4	3.1	2.4	0.4	0.5	0.7
STP4	49.0	52.8	46.5	6.4	8.7	12.5
STP7	0.0	0.1	0.0	0.0	0.0	0.0
STP9	0.1	0.4	0.2	3.0	6.1	7.1
STP15	2.1	1.5	2.0	1.9	1.5	3.3
STP16	10.6	10.8	11.2	0.4	0.9	3.0
STP17	2.8	2.5	2.4	0.4	0.1	0.1
STP20	33.6	21.8	23.2	0.5	0.3	0.7
STP23	10.8	7.9	8.4	65.6	103.4	124.0
STP24	9.6	8.2	9.9	32.1	50.5	56.3
STP25	14.7	15.2	16.9	2.5	2.3	2.9
STP26	1.6	1.0	1.8	0.1	0.2	0.2
STP30	26.5	17.7	20.2	0.8	1.4	1.3
STP31	0.0	0.0	0.0	0.0	0.0	0.0
STP33	0.6	0.4	0.5	0.3	0.2	0.6
PIP1-2	265.9	197.6	220.5	291.7	282.6	209.4
PIP1-3	136.4	82.6	88.5	551.3	560.6	393.6
PIP1-9	15.7	12.6	11.8	308.7	327.6	275.6
PIP1-11	77.9	66.6	69.8	135.5	117.7	76.9
PIP1-13	7.2	5.4	5.5	14.1	14.8	7.9
PIP2-1	136.1	126.6	138.4	731.7	650.9	527.8
PIP2-3	45.9	26.0	31.3	533.4	423.3	266.8
PIP2-9	57.3	38.4	42.3	171.7	137.8	84.2
PIP2-12	0.2	0.1	0.1	0.2	0.5	0.6
PIP2-13	4.5	4.8	3.2	82.8	70.4	58.9
PIP2-14	2.8	3.2	2.6	84.4	74.9	74.5

PIP2-15	0.8	1.0	0.5	8.6	8.4	8.0
PIP2-16	0.4	0.1	0.1	7.8	6.8	6.4
PIP2-17	111.7	110.4	113.3	32.6	29.9	30.6
PIP2-19	0.9	0.4	0.7	0.5	0.2	1.0
PIP2-21	91.5	49.7	61.8	235.0	203.1	144.5
SYP111	0.5	0.4	1.0	2.2	4.0	3.0
SYP112	0.2	0.1	0.1	0.0	0.1	1.0
SYP123	0.1	0.1	0.1	0.0	0.1	0.1
SYP131	2.0	2.1	2.1	1.4	2.0	2.0
SYP21	12.6	11.1	12.5	12.1	13.2	15.1
SYP31	11.1	7.7	8.0	10.8	13.2	21.0
SYP41	15.0	14.8	15.1	24.3	24.7	26.1
VTI11	14.1	14.7	14.9	31.9	31.6	30.0
VTI14	22.0	26.5	26.6	32.7	35.7	34.8
GOS11	11.6	8.6	10.1	6.6	11.9	13.1
GOS12	4.6	4.8	5.0	9.4	12.0	11.8
MEMB11	3.0	3.6	3.1	4.6	4.8	5.1
NPSN11	2.1	2.2	2.0	0.6	1.3	1.7
NPSN12	4.8	3.4	3.7	4.1	4.5	7.0
SEC20	6.5	6.8	6.5	12.2	13.7	12.2
SYP51	18.3	20.6	19.2	16.1	20.9	21.2
SYP61	19.6	15.2	13.8	7.5	11.5	13.3
SYP71	12.9	16.7	15.8	10.6	10.7	9.9
SFT11	12.8	13.7	15.8	22.2	24.0	18.1
USE11	88.0	106.8	110.8	71.6	71.7	74.8
VAMP711	27.0	28.9	28.3	39.6	36.1	36.6
VAMP721	32.8	31.5	34.8	36.8	43.3	36.9
VAMP724	3.2	2.0	2.2	2.7	3.9	4.2
VAMP725	32.8	31.5	34.8	36.8	43.3	36.9
VAMP727	24.5	32.1	32.3	39.5	37.6	36.7
YKT61	10.4	13.3	12.0	24.1	25.1	20.1

Supplementary Table S6: TPM values of different genes in *Ananas comosus.*

	leaf			nectary		
**gene name**	**TPM1**	**TPM2**	**TPM3**	**TPM1**	**TPM2**	**TPM3**
SPS1	45.2	22.8	24.8	11.7	33.5	33.9
SPS2	72.4	41.3	44.5	3.6	5.5	6.0
SPS4	0.0	0.1	0.1	3.2	5.4	5.7
SPS5	0.3	0.2	0.3	0.6	0.9	1.1
NINV1	41.2	25.6	26.9	13.5	21.5	25.1
CWINV1	5.7	4.1	4.0	47.6	95.7	106.4
CWINV2	2.4	1.8	2.0	3.1	3.6	2.9
VINV1	222.8	103.6	113.7	12.4	27.6	28.7
SUS1	466.7	260.1	277.0	279.6	435.2	476.7
SUS1.1	2.9	2.1	2.0	88.7	115.5	138.6
SUS3	722.7	461.6	489.0	128.6	206.8	218.7
SUS6	0.2	0.3	0.1	6.6	12.4	11.2
SWEET1	68.1	43.3	47.6	2.6	6.8	5.8
SWEET2	1.2	1.0	1.0	1.1	1.7	1.4
SWEET3	56.3	31.3	41.5	1.4	3.2	2.3
SWEET4	1.1	0.4	0.7	4.5	7.8	6.7
SWEET5	55.0	40.4	47.8	216.6	318.2	300.3
SWEET6	0.0	0.0	0.1	25.2	44.8	38.9
SWEET7	0.0	0.0	0.0	1.6	3.5	3.2
SWEET9	0.0	0.1	0.0	0.4	0.0	0.0
SWEET10	0.0	0.0	0.0	0.3	0.4	0.4
SWEET11	112.1	85.0	89.9	32.2	54.3	50.2
SWEET13	0.4	0.3	0.3	0.7	0.8	0.8
SWEET14	7.1	7.1	8.6	1.9	1.9	1.4
SWEET15	0.1	0.1	0.2	34.5	64.1	55.2
SWEET16	0.2	0.1	0.2	1.6	2.3	2.2
SUT1	24.6	13.3	15.3	274.5	527.1	496.3
SUT2	5.7	2.6	2.9	3.0	7.5	7.5
SUT4	2.8	1.3	1.4	4.0	13.5	12.4
STP1	0.3	0.3	0.3	236.2	579.8	489.1
STP2	0.6	0.3	0.3	0.8	0.4	0.4
STP3	0.0	0.0	0.0	0.1	0.1	0.0
STP4	7.5	5.0	5.1	0.5	0.6	0.6
STP5b	0.0	0.1	0.1	10.9	24.3	21.0
STP5d	6.3	4.2	3.6	3.6	7.6	7.0
STP5e	0.5	0.1	0.4	0.4	0.2	0.3
STP6a	0.1	0.0	0.0	1.7	3.7	3.6
STP6b	40.1	14.6	17.8	10.9	30.1	33.0
STP7	0.0	0.0	0.0	3.6	7.1	5.5
STP8	0.5	0.3	0.3	22.7	44.9	38.4
STP10	0.1	0.0	0.0	18.2	41.2	38.6
STP13	27.7	19.5	22.3	52.7	63.9	65.3
STP23	1.1	0.9	1.0	1.5	1.4	1.6
STP25	1.5	1.2	1.2	17.0	15.0	17.7
STP26	9.8	7.1	8.5	15.2	14.6	16.7
PIP1-1	767.5	311.0	356.0	469.3	545.5	141.3
PIP1-2	70.6	20.9	25.6	709.1	794.9	173.4
PIP1-3	3.9	1.4	2.0	26.4	36.3	6.4
PIP1-4	16.6	8.8	11.0	162.6	185.8	110.1
PIP2-1	8.6	3.2	4.5	175.6	159.8	121.4
PIP2-2	30.1	25.2	25.3	199.8	208.7	222.7
PIP2-3	0.7	0.4	0.5	12.9	11.0	8.4
PIP2-4	273.9	209.4	204.1	1072.5	994.3	796.2
PIP2-5	766.3	366.5	425.0	106.6	98.8	43.6
SYP111	0.4	0.3	0.2	2.7	5.8	4.9
SYP112	0.6	0.5	1.0	5.1	3.2	2.1
SYP121	139.2	143.5	103.6	34.5	31.3	32.4
SYP123	0.0	0.0	0.1	91.4	123.2	114.5
SYP131	0.1	0.1	0.1	1.9	2.8	2.5
SYP21	53.2	31.7	30.6	29.9	50.8	53.5
SYP22	13.2	8.1	9.7	15.6	17.4	15.3
SYP31	5.6	3.1	4.1	12.0	20.4	19.5
SYP41	0.3	0.2	0.4	5.5	15.7	13.1
SYP42	3.6	2.6	2.6	4.1	8.6	8.4

NPSN12	25.6	30.4	30.1	98.2	60.1	63.3
SEC20	10.3	8.6	9.2	14.4	19.6	18.0
BET11	53.7	46.9	47.7	129.8	149.3	139.5
SYP51	10.7	10.1	10.6	29.7	29.3	26.2
SYP61	7.3	6.6	7.2	18.0	21.8	20.1
SYP71	34.0	28.0	28.2	83.5	85.0	91.1
SFT11	10.4	16.0	15.7	57.0	37.3	34.0
USE11	3.8	5.2	5.2	13.0	12.3	9.8
SNAP29	13.9	9.9	10.3	34.8	68.3	56.7
SNAP30	0.5	0.4	0.5	3.0	2.8	2.5
SNAP33	10.0	5.5	6.9	1.6	1.8	2.9
VAMP711	125.5	129.7	127.0	193.5	68.3	181.6
VAMP712	33.0	22.7	23.9	34.4	53.4	52.9
VAMP721	9.9	5.7	6.8	8.9	18.0	17.6
VAMP724	11.0	10.3	9.8	25.9	26.6	30.1
VAMP725	13.6	10.3	11.1	20.5	31.7	34.0
VAMP727	13.5	9.9	9.5	10.7	13.9	17.6
YKT61	9.2	6.5	6.4	22.1	27.8	27.8
SEC221	9.7	5.7	6.3	13.2	29.5	26.6

Supplementary Table S7: Differential expression log2 ratios of different genes in *Nicotiana tabacum.*

The significant difference is indicated by asterisks (differential expression *p*-value < 0.05).

Genes	Log2 ratio	Genes	Log2 ratio	Genes	Log2 ratio
SPSB	6.4*	*SWEET10*	-1.5*	*PIP2-15*	3.5*
SPSC	-2.3*	*SWEET11*	-4.3*	*PIP2-16*	5.0*
NINV1	-0.8*	*SWEET12*	4.0*	*PIP2-17*	-1.8*
NINV3	1.6*	*SUT1*	-0.9*	*PIP2-19*	0.0
NINV4	0.3	*SUT2*	-0.8*	*PIP2-21*	1.6*
NINV5	0.6*	*SUT4*	2.8*	*SYP111*	2.2*
NINV6	3.1*	*STP3*	-2.6*	*SYP112*	1.1
NINV12	-3.7*	*STP4*	-2.4*	*SYP123*	-0.5
NINV13	3.1*	*STP7*	-0.7	*SYP131*	-0.2
NINV15	1.2*	*STP9*	4.5*	*SYP21*	0.2
NINV16	-3.7*	*STP15*	0.3	*SYP31*	0.9*
NINV19	1.0*	*STP16*	-3.0*	*SYP41*	0.8*
VINV1	1.1*	*STP17*	-3.5*	*VTI11*	1.2*
VINV2	1.4*	*STP20*	-5.7*	*VTI14*	0.5
VINV3	-1.0*	*STP23*	3.4*	*GOS11*	0.1
CWINV2	-0.3	*STP24*	2.3*	*GOS12*	1.2*
CWINV5	-6.3*	*STP25*	-2.5*	*MEMB11*	0.6*
CWINV6	2.8	*STP26*	-3.1*	*NPSN11*	-0.8*
SUS1	8.6*	*STP30*	-4.2*	*NPSN12*	0.4*
SUS2	2.3*	*STP31*	-0.3	*SEC20*	1.0*
SUS3	-0.2	*STP33*	-0.5	*SYP51*	0.0
SUS5	2.4*	*PIP1-2*	0.2	*SYP61*	-0.6*
SUS6	0.5*	*PIP1-3*	2.3*	*SYP71*	-0.5
SUS7	2.5*	*PIP1-9*	4.6*	*SFT11*	0.7
SWEET1	1.5*	*PIP1-11*	0.6*	*USE11*	-0.4
SWEET1a	-0.2	*PIP1-13*	1.0*	*VAMP711*	0.5
SWEET2a	-0.2	*PIP2-1*	2.3*	*VAMP721*	0.3
SWEET2b	-1.4*	*PIP2-3*	3.6*	*VAMP724*	0.6*
SWEET4	8.3*	*PIP2-9*	1.5*	*VAMP725*	0.3
SWEET5	-0.6	*PIP2-12*	1.8*	*VAMP727*	0.4
SWEET7	8.9*	*PIP2-13*	4.1*	*YKT61*	1.0*
SWEET9	12.8*	*PIP2-14*	4.8*		

Supplementary Table S8: Differential expression log2 ratios of different genes in *Ananas comosus*.

The significant difference is indicated by asterisks (differential expression *p*-value < 0.05).

Genes	Log2 ratio	Genes	Log2 ratio	Genes	Log2 ratio
SPS1	-1.2*	SUT4	1.4*	SYP121	-2.9*
SPS2	-4.3*	STP1	9.3*	SYP123	9.9*
SPS4	5.1*	STP2	-0.5	SYP131	3.6*
SPS5	0.6*	STP3	0.4	SYP21	-0.8*
NINV1	-1.6*	STP4	-4.3*	SYP22	-0.3
VINV1	-3.6*	STP5b	6.9*	SYP31	1.0*
CWINV1	3.2*	STP5d	-0.6*	SYP41	4.3*
CWINV2	-0.4	STP5e	-0.9	SYP42	0.3
SUS1	-0.7*	STP6a	5.0*	NPSN12	0.4
SUS1.1	4.6*	STP6b	-0.9*	SEC20	-0.1
SUS3	4.6*	STP7	9.6*	BET11	0.5*
SUS6	-2.6*	STP8	5.6*	SYP51	0.5*
SWEET1	-4.4*	STP10	8.4*	SYP61	0.5*
SWEET2	-0.6*	STP13	0.4*	SYP71	0.6*
SWEET3	-5.2*	STP23	-0.3	SFT11	0.6
SWEET4	2.2*	STP25	2.7*	USE11	0.3
SWEET5	1.6*	STP26	-0.1	SNAP29	1.2*
SWEET6	8.7*	PIP1-1	-1.3*	SNAP30	1.6*
SWEET7	5.3*	PIP1-2	2.8*	SNAP33	-2.8*
SWEET9	0.3	PIP1-3	2.2*	VAMP711	-0.5
SWEET10	4.5*	PIP1-4	2.7*	VAMP712	-0.2
SWEET11	-2.1*	PIP2-1	3.9*	VAMP721	0.0
SWEET13	0.4*	PIP2-2	2.0*	VAMP724	0.4*
SWEET14	-3.1*	PIP2-3	3.4*	VAMP725	0.3
SWEET15	7.5*	PIP2-4	1.1*	VAMP727	-0.6*
SWEET16	2.6*	PIP2-5	-3.6*	YKT61	0.8*
SUT1	3.6*	SYP111	2.8*	SEC221	0.7*
SUT2	-0.3	SYP112	1.3*		

Supplementary Table S9: Gene IDs of *Arabidopsis thaliana* genes. Genes are divided into the following groups: SPS, INV, SUS, SUT, SWEET, STP, PIP, SNARE. The genes can be found with the gene ID in the NCBI database.

Gene common name	Gene ID	Gene common name	Gene ID	Gene common name	Gene ID
AtSPS1F	At5g20280	*AtSTP3*	AT5G61520	*AtSYP131*	AT3G03800
AtSPS2F	At5g11110	*AtSTP4*	AT3G19930	*AtSYP132*	AT5G08080
AtSPS3F	At1g04920	*AtSTP5*	AT1G34580	*AtSYP21*	AT5G16830
AtSPS4F	At4g10120	*AtSTP6*	AT3G05960	*AtSYP22*	AT5G46860
AtNINV1	At1g56560	*AtSTP7*	AT4G02050	*AtSYP23*	AT4G17730
AtCWINV1	At3g13790	*AtSTP8*	AT5G26250	*AtSYP31*	AT5G05760
AtCWINV2	At3g52600	*AtSTP9*	AT1G50310	*AtSYP32*	AT3G24350a
AtCWINV4	At2g36190	*AtSTP10*	AT3G19940	*AtSYP41*	AT5G26980
AtCWINV5	At3g13784	*AtSTP11*	AT5G23270	*AtSYP42*	AT4G02195
AtCWINV6	At5g11920	*AtSTP12*	AT4G21480	*AtSYP43*	AT3G05710
AtSus1	At5g20830	*AtSTP13*	AT5G26340	*AtSYP81*	AT1G51740
AtSus2	At5g49190	*AtSTP14*	AT1G77210	*AtVTI11*	AT5G39510
AtSus3	At4g02280	*AtSUC1*	AEE35247.1	*AtVTI12*	AT1G26670
AtSus4	At3g43190	*AtSUC2*	AEC05635.1	*AtVTI13*	AT3G29100
AtSus5	At5g37180	*AtSUT4*	NP_172467.1	*AtVTI14*	AT5G39630
AtSus6	At1g73370	*AtPIP1-1*	AT3G61430	*AtGOS11*	AT1G15880
AtSWEET1	At1g21460	*AtPIP1-2*	AT2G45960	*AtGOS12*	AT2G45200
AtSWEET2	At3g14770	*AtPIP1-3*	AT1G01620	*AtMEMB11*	AT2G36900
AtSWEET3	At5g53190	*AtPIP1-4*	AT4G00430	*AtMEMB12*	AT5G50440
AtSWEET4	At3g28007	*AtPIP1-5*	AT4G23400	*AtNPSN11*	AT2G35190
AtSWEET5	At5g62850	*AtPIP2-1*	AT3G53420	*AtNPSN12*	AT1G48240
AtSWEET6	At1g66770	*AtPIP2-2*	AT2G37170	*AtNPSN13*	AT3G17440
AtSWEET7	At4g10850	*AtPIP2-3*	AT2G37180	*AtSEC20*	AT3G24315
AtSWEET8	At5g40260	*AtPIP2-4*	AT5G60660	*AtBET11*	AT3G58170
AtSWEET9	At2g39060	*AtPIP2-5*	AT3G54820	*AtSYP51*	AT1G16240
AtSWEET10	At5g50790	*AtPIP2-6*	AT2G39010	*AtSYP52*	AT1G79590
AtSWEET11	At3g48740	*AtPIP2-7*	AT4G35100	*AtSYP61*	AT1G28490
AtSWEET12	At5g23660	*AtPIP2-8*	AT2G16850	*AtSYP71*	AT3G09740
AtSWEET13	At5g50800	*AtSYP111*	AT1G08560	*AtSYP72*	AT3G45280
AtSWEET14	At4g25010	*AtSYP112*	AT2G18260	*AtSYP73*	AT3G61450
AtSWEET15	At5g13170	*AtSYP121*	AT3G11820	*AtSFT11*	AT4G14600
AtSWEET16	At3g16690	*AtSYP122*	AT3G52400	*AtSFT12*	AT1G29060
AtSWEET17	At4g15920	*AtSYP123*	AT4G3330	*AtUSE11*	AT1G54110
AtSTP1	AT1G11260	*AtSYP124*	AT1G61290	*AtUSE12*	AT3G55600
AtSTP2	AT1G07340	*AtSYP125*	AT1G11250	*AtSNAP29*	AT5G07880

**Gene common name**	**Gene ID**
AtSNAP30	AT1G13890
AtSNAP33	AT5G61210
AtVAMP711	AT4G32150
AtVAMP712	AT2G25340
AtVAMP713	AT5G11150
AtVAMP714	AT5G22360
AtVAMP721	AT1G04750
AtVAMP722	AT2G33120
AtVAMP724	AT4G15780
AtVAMP725	AT2G32670
AtVAMP726	AT1G04760
AtVAMP727	AT3G54300
AtYKT61	AT5G58060
AtYKT62	AT5G58180
SEC221	AT1G11890

129

Supplementary Table S10: Gene IDs of *Nicotiana tabacum* genes.
Genes are divided into the following groups: SPS, INV, SUS, SUT, SWEET, STP, PIP, SNARE. The genes can be found with the gene ID in the NCBI database and with another gene ID in the *N. tabacum* genome (Edwards et al., 2017). The genes were named using the following references: Chen et al., 2005; Wang et al., 2015; Pfister et al, 2017; Ahmed et al., 2020; Xu et al., 2022; Cheng et al., 2023.

Gene common name	Gene ID	Gene ID Genome
NtSPSB	DQ213015	0000401g0120
NtSPSC	DQ213014	0000009g0480
NtNINV1	LOC107778980	0003838g0090
NtNINV3	LOC107819026	0001082g0080
NtNINV4	LOC107816919	0000962g0090
NtNINV5	LOC107768486	0002561g0010
NtNINV6	LOC107781195	0000589g0010
NtNINV8	LOC107768486	0002561g0010
NtNINV12	LOC107797521	0000283g0090
NtNINV13	LOC107781195	0000589g0010
NtNINV14	LOC107778980	0003838g0090
NtNINV15	LOC107826453	0000365g0230
NtNINV16	LOC107797521	0000283g0090
NtNINV19	LOC107788023	0000303g0260
NtNINV20	LOC107768486	0002561g0010
NtCWINV2	AF376773.1	0011403g0010
NtCWINV5	HM022265.1	0001295g0210
NtCWINV6	ADI70683.1	0002654g0150
NtVINV1	LOC107770131	0001383g0030
NtVINV2	LOC107810155	0001780g0120
NtVINV3	LOC107807993	0000993g0100
NtSus1	LOC107783276	0000073g0400
NtSus2	LOC107775654	0002280g0010
NtSus3	LOC107804066	0000170g0050
NtSus5	LOC107775584	0000116g0360
NtSus6	LOC107766404	0000483g0250
NtSus7	LOC107789475	0001180g0180
NtSWEET1	LOC107806439	0000445g0160.1
NtSWEET1a	LOC107787259	0004095g0060.1
NtSWEET2A	XM_016629722.1	0003746g0030
NtSWEET2B	XM_016634622.1	0001140g0150
NtSWEET4	LOC107804242	0000673g0010.1
NtSWEET5	LOC107797107	0000003g0510.1
NtSWEET7	LOC107794016	0002367g0130.1

Gene common name	Gene ID	Gene ID Genome
NtSWEET9	LOC107793141	0004802g0020
NtSWEET10	LOC107788219	0001332g0120.1
NtSWEET11	LOC107760004	0000033g0010.1
NtSWEET12	LOC107760005	0000033g0030.1
NtSTP3	XP_016474706.1	0003465g0030
NtSTP4	XP_016494599.1	0000190g0030
NtSTP7	XP_016476713.1	0002234g0120
NtSTP9	XP_016484433.1	0004425g0040
NtSTP15	XP_016475527.1	0002387g0060
NtSTP16	XP_016488213.1	0000069g0190
NtSTP17	XP_016479727.1	0000232g0280
NtSTP20	XP_016434630.1	0001267g0050
NtSTP23	XP_016435043.1	0000416g0010
NtSTP24	XP_016493725.1	0001671g0060
NtSTP25	XP_016446212.1	0000012g0180
NtSTP26	XP_016483827.1	0001004g0170
NtSTP30	XP_016500201.1	0000795g0070
NtSTP31	XP_016458581.1	0000102g0190
NtSTP33	XP_016515629.1	0000559g0110
NtSUT1	MF140390.1	0000159g0100
NtSUT2	LC497468.1	0003062g0010
NtSUT4	AB539539.1	0000377g0120
NtPIP1-2	XP_016508253.1	0001615g0140
NtPIP1-3	AAB04757.1	0003043g0010
NtPIP1-9	NP_001312921.1	0000737g0120
NtPIP1-11	XP_016515710.1	0000846g0060
NtPIP1-13	XP_016510215.1	0000583g0150
NtPIP2-1	AAL33586.1	0000283g0420
NtPIP2-3	NP_001312414.1	0003914g0040
NtPIP2-9	NP_001312511.1	0000575g0130
NtPIP2-12	NP_001312276.1	0001192g0080
NtPIP2-13	NP_001312334.1	0009795g0010
NtPIP2-14	XP_016486700.1	0000101g0120
NtPIP2-15	NP_001312333.1	0009795g0020

Gene common name	Gene ID	Gene ID Genome
NtPIP2-16	XP_016513533.1	0000101g0110
NtPIP2-17	NP_001312464.1	0000650g0260
NtPIP2-19	NP_001313208.1	0000106g0170
NtPIP2-21	NP_001311765.1	0000181g0120
NtSYP111	XP_016479641.1	0000302g0040
NtSYP112	XP_016489059.1	0000178g0230
NtSYP123	XP_016462928.1	0000635g0160
NtSYP131	XP_016510458.1	0004274g0010
NtSYP21	XP_016506496.1	0000109g0090
NtSYP31	XP_016462826.1	0000125g0090
NtSYP41	XP_016443453.1	0002715g0150
NtVTI11	XP_016500539.1	0001997g0110
NtVTI14	NP_001312652.1	0001195g0020
NtGOS11	XP_016486928.1	0002503g0100
NtGOS12	XP_016482025.1	0000859g0340
NtMEMB11	XP_016454786.1	0001741g0020
NtNPSN11	XP_016440521.1	0001981g0010
NtNPSN12	XP_016513453.1a	0000895g0240
NtSEC20	XP_016474547.1	0001041g0140
NtSYP51	XP_016487954.1	0001160g0040
NtSYP61	XP_016456479.1	0000151g0150
NtSYP71	XP_016478104.1	0001192g0040
NtSFT11	XP_016464993.1	0001984g0080
NtUSE11	XP_016471202.1	0000630g0070
NtVAMP711	XP_016451055.1	0000109g0410
NtVAMP721	XP_016433615.1	0002085g0030
NtVAMP724	XP_016443055.1	0000960g0120
NtVAMP725	XP_016507558.1	0002085g0030
NtVAMP727	XP_016457065.1	0002586g0070
NtYKT61	XP_016496169.1	0001094g0120

Supplementary Table S11: Gene IDs of *Oryza sativa* genes.
Genes are divided into the following groups: SPS, INV, SUS, SUT, SWEET, STP, PIP, SNARE. The genes can be found with the gene ID in the NCBI database.

Gene common name	Gene ID
OsSPS1	Q0JGK4
OsSPS2	B7F7B9.2
OsSPS3	Q67WN8
OsSPS4	Q6ZHZ1
OsSPS5	Q53JI9
OsNINV1	Os02g34560
OsNINV2	Os04g35280
OsNINV3	Os11g07440
OsNINV4	Os02g03320
OsNINV5	Os02g32730
OsNINV6	Os04g33490
OsNINV7	Os01g22900
OsNINV8	Os03g20020
OsCWINV1	Os03g52560
OsCWINV2	Os04g33720
OsCWINV3	Os04g33740
OsCWINV4	Os02g33110
OsCWINV5	Os01g73580
OsCWINV6	Os04g56930
OsCWINV7	Os04g56920
OsCWINV8	Os09g08120
OsCWINV9	Os09g08072
OsVINV1	Os02g01590
OsVINV2	Os04g45290
OsSus1	NP_001389102.1
OsSus2	NP_001389706.1
OsSus3	NP_001390086.1
OsSus4	NP_001389082.1
OsSus5	NP_001406164.1
OsSus6	NP_001388945.1
OsSWEET1A	AK099531
OsSWEET1B	AK063475
OsSWEET2A	AK104255
T2B	AK059965
OsSWEET3A	NP_001407211.1
OsSWEET3B	XP_015642315.1
OsSWEET4	AK071676
OsSWEET5	AK069614
OsSWEET6A	NP_001415004.1
OsSWEET6B	AK099440
OsSWEET7A	Q0J361.2
OsSWEET7B	XP_015611083.1
OsSWEET7C	XP_015619157.1
OsSWEET7D	B9G2E6.3
OsSWEET7E	A3BWJ9.2
OsSWEET11	AK106127
OsSWEET12	AK109114
OsSWEET13	CI437556
OsSWEET14	AK101913
OsSWEET15	AK103266
OsSWEET16	CI149956
OsSTP1	Os04g37980.1
OsSTP2	Os03g39710.1
OsSTP3	Os07g01560.1
OsSTP4	Os03g11900.1
OsSTP8	Os01g38670.1
OsSTP10	Os02g36414.1
OsSTP11	Os02g36440.1
OsSTP13	Os03g01170.1
OsSTP14	Os04g37970.1
OsSTP15	Os04g37990.1
OsSTP16	Os04g38010.1
OsSTP17	Os04g38026.1
OsSTP18	Os04g38220.1
OsSTP19	Os06g04900.1
OsSTP20	Os07g03910.1
OsSTP21	Os07g03960.1
OsSTP26	Os09g24924.1
OsSTP28	Os11g38160.1
OsSUT1	BAA24071
OsSUT2	AB091672
OsSUT3	BAB68368
OsSUT4	AB091673
OsSUT5	AB091674
OsPIP1-1	BAD28398
OsPIP1-2	Os04g47220
OsPIP1-3	BAD22920
OsPIP2-1	BAC15868
OsPIP2-2	BAD23735
OsPIP2-3	CAD41442
OsPIP2-4	BAC16113
OsPIP2-5	BAC16116
OsPIP2-6	CAE05002
OsPIP2-7	BAD46581
OsPIP2-8	AAP44741
OsSYP81	XP_015635852.1
OsSYP32	XP_015617756.1
OsSYP41	XP_015641289.1
OsSYP21	XP_015622380.1
OsSYP111	XP_015631526.1
OsSYP121	XP_015628438.1
OsSYP123	XP_015624037.1
OsSYP132	XP_015647004.1
OsSEC20	XP_015623272.1
OsMemB11	XP_015633085.1
OsGOS11	XP_015611820.1
OsGOS12	XP_015627527.1
OsVTI11	XP_015621845.1
OsNPSN11	XP_015642684.1
OsNPSN12	XP_015646134.1
OsUSE12	XP_015651428.1
OsBSA14A	XP_015648254.1
OsSFT11	XP_015645414.1
OsSYP61	XP_015625156.1
OsSYP71	XP_015639751.1

Gene common name	Gene ID
OsSNAP29	NP_001403563.1
OSYKT61	NP_001393116.1
OsVAMP711	XP_015641118.1
OsVAMP714	XP_015614986.1
OsVAMP724	XP_015645337.1
OsVAMP727	XP_015649386.1
OsVAMP725	XP_015647709.1

Supplementary Table S12: Gene IDs of *Ananas comosus* genes. Genes are divided into the following groups: SPS, INV, SUS, SUT, SWEET, STP, PIP, SNARE. The genes can be found with the gene ID in the NCBI database and in the *A. comosus* genome (Ming et al., 2015). The genes were named using the following references: Guo et al., 2018; Zhu and Ming, 2019; Wu et al., 2022; Gao et al., 2022; Fakher et al., 2022.

Gene common name	Gene ID
AcSPS1	LOC109724677
AcSPS2	LOC109712530
AcSPS4	LOC109725728
AcSPS5	LOC109726629
AcNINV1	LOC109726732
AcNINV2	LOC109707584
AcNINV3	LOC109712448
AcNINV4	LOC109728768
AcNINV5	LOC109707134
AcCWINV1	LOC109725952
AcCWINV2	LOC109725753
AcVINV1	LOC109727167
AcSus1	LOC109714349
AcSus1.1	LOC109717643
AcSus3	LOC109726980
AcSus6	LOC109719969
AcSWEET1	LOC109717722
AcSWEET2	LOC109717521
AcSWEET3	LOC109716017
AcSWEET4	LOC109710588
AcSWEET5	LOC109717849
AcSWEET6	LOC109716724
AcSWEET7	LOC109722106
AcSWEET9	LOC109723368
AcSWEET10	LOC109725827
AcSWEET11	LOC109723713
AcSWEET13	LOC109726183
AcSWEET14	LOC109710404
AcSWEET15	LOC109709226
AcSWEET16	LOC109720315
AcSTP1	LOC109712863
AcSTP2	LOC109708566
AcSTP3	LOC109708611
AcSTP4	LOC109721830
AcSTP5b	LOC109718670
AcSTP5c	LOC109718670
AcSTP5d	LOC109725394
AcSTP5e	LOC109725502
AcSTP6a	LOC109713301
AcSTP6b	LOC109715036
AcSTP7	LOC109718125
AcSTP8	LOC109724884
AcSTP10	LOC109707304
AcSTP13	LOC109713738
AcSTP23	LOC109726972
AcSTP25	LOC109708573
AcSTP26	LOC109706529
AcSUT1	LOC109720885
AcSUT2	LOC109727628
AcSUT4	LOC109718518
AcPIP1-1	LOC109708083
AcPIP1-2	LOC109703497
AcPIP1-3	LOC109721095
AcPIP1-4	LOC109727137
AcPIP2-1	LOC109707937
AcPIP2-2	LOC109715516
AcPIP2-3	LOC109713634
AcPIP2-4	LOC109708149
AcPIP2-5	LOC109713056
AcSYP111	LOC109724005
AcSYP112	LOC109705538
AcSYP121	LOC109713508
AcSYP123	LOC109710368
AcSYP131	LOC109715908
AcSYP21	LOC109727640
AcSYP22	LOC109707486
AcSYP31	LOC109710966
AcSYP1	LOC109713584
AcSYP42	LOC109727303
AcNPSN12	LOC109727331
AcSEC20	LOC109723551
AcBET11	LOC109723641
AcSYP51	LOC109722298
AcSYP61	LOC109723183
AcSYP71	LOC109710984
AcSFT11	LOC109727331
AcUSE11	LOC109723551
AcSNAP29	LOC109721766
AcSNAP30	LOC109712973
AcSNAP33	LOC109708842
AcVAMP711	LOC109711499
AcVAMP712	LOC109710881
AcVAMP721	LOC109715685
AcVAMP724	LOC109718244
AcVAMP725	LOC109710494
AcVAMP727	LOC109724556
AcYKT61	LOC109725941
AcSEC221	LOC109709435
